# Contributions to Obstetric Pathology and Practice

**Published:** 1853-08

**Authors:** 


*Contributions to Obstetric Pathology and Practice.* By JAMES Y. SIMPSON, M. D., Prof. of Midwifery in the University of Edinburgh.

These contributions appeared first in the Edinburgh "Monthly Journal of Medical Sciences." The collection embraces "Chloroform in Infantile Convulsions;" "Turning as a substitute for Craniotomy;" "Albuminuria in Puerperal and Infantile Convulsions;" and sundry other papers. Like Dr. Simpson's previous contributions they present evidences of an active, even restless mind, leading to original thinking, and not unfrequently to that farther stretch of activity, called hobby-riding. The paper on chloroform is a valuable and practical one. Those who originally applied chloroform to serious convulsive disease deserve credit; and Buffalo has the earliest claim on this continent, in a case reported in this Journal by Prof. J. P. White, six months after the announcement of its anæsthetic powers. To the conclusions on turning as a substitute for craniotomy, most obstetricians will object. In the fact that the head presentation is the one ordained by nature, we see that it is best adapted to the relative diameters of the head and the pelvis. Turning disturbs and reverses those diameters; and we can only gain the use of the child's body and neck as a means of traction, while we lose that exact adaptation of part to part, which we have in the ordinary presentation.

